# Homologs of *LEAFY* and *UNUSUAL FLORAL ORGANS* Promote the Transition From Inflorescence to Floral Meristem Identity in the Cymose *Aquilegia coerulea*

**DOI:** 10.3389/fpls.2019.01218

**Published:** 2019-10-04

**Authors:** Bharti Sharma, Clara Meaders, Damien Wolfe, Lynn Holappa, Cristina Walcher-Chevillet, Elena M. Kramer

**Affiliations:** ^1^Department of Biological Sciences, California State Polytechnic University, Pomona, CA, United States; ^2^Department of Organismic and Evolutionary Biology, Harvard University, Cambridge, MA, United States; ^3^Department of Ecology and Evolutionary Biology, Cornell University, Ithaca, NY, United States

**Keywords:** *Aquilegia*, inflorescence structure, floral meristem identity, *LEAFY*, *UNUSUAL FLORAL ORGANS*

## Abstract

Homologs of the transcription factor *LEAFY* (*LFY*) and the F-box family member *UNUSUAL FLORAL ORGANS* (*UFO*) have been found to promote floral meristem identity across diverse dicot model systems. The lower eudicot model *Aquilegia* produces cymose inflorescences that are independently evolved from the well-studied cymose models *Petunia* and tomato. We have previously characterized the expression pattern of the *Aquilegia* homolog *AqLFY* but in the current study, we add expression data on the two *UFO* homologs, *AqUFO1* and *2*, and conduct virus-induced gene silencing of all the loci. Down-regulation of *AqLFY* or *AqUFO1* and *2* does not eliminate floral meristem identity but, instead, causes the transition from inflorescence to floral identity to become gradual rather than discrete. Inflorescences in down-regulated plants generate several nodes of bract/sepal chimeras and, once floral development does commence, flowers initiate several whorls of sepals before finally producing the wildtype floral whorls. In addition, silencing of *AqUFO1/2* appears to specifically impact petal identity and/or the initiation of petal and stamen whorls. In general, however, there is no evidence for an essential role of *AqLFY* or *AqUFO1/2* in transcriptional activation of the B or C gene homologs. These findings highlight differences between deeply divergent dicot lineages in the functional conservation of the floral meristem identity program.

## Introduction

The generation of inflorescence architecture reflects a complex interplay between two meristem identity programs (reviewed Bartlett and Thompson, 2014). By varying the phyllotactic and temporal patterns of inflorescence meristem (IM) and floral meristem (FM) identity expression, plants can generate an enormous diversity of branching patterns. The genetic basis of these two identity programs was first investigated in several model systems that produce racemes. In this monopodial architecture, the terminal IM retains indeterminacy while lateral meristems may immediately express determinate FM identity or, in the case of branched inflorescences, the expression of FM identity may be delayed by one or more branching orders. Studies of the racemose *Arabidopsis thaliana* and *Antirrhinum majus* revealed homologous genes that control IM and FM identity: IM identity being primarily promoted by orthologs of the *A. thaliana* gene *TERMINAL FLOWER* (*TFL*; Alvarez et al., 1992) and *FRUITFULL* (*FUL*; Ferrandiz et al., 2000), while FM identity is promoted by orthologs of the *A. thaliana* gene *LEAFY* (*LFY*; Weigel et al., 1992) and *APETALA1* (*AP1*; Bowman et al., 1993). *LFY* is further required to activate all of the genes that confer floral organ identity (Weigel and Meyerowitz, 1993), as described by the ABC model (Coen and Meyerowitz, 1991). Interestingly, each class of ABC gene is activated by a distinct LFY-containing complex. The A class and FM identity gene *AP1* can be up-regulated by LFY alone (Parcy et al., 1998). In contrast, the B class gene *APETALA3*, which confers petal and stamen identity, requires the presence of a co-factor, the F-box protein UNUSUAL FLORAL ORGANS (Lee et al., 1997). Finally, the stamen and carpel identity C class gene *AGAMOUS* is activated by LFY together with the homeodomain protein WUSCHEL (Lohmann et al., 2001).

**Figure 1:**
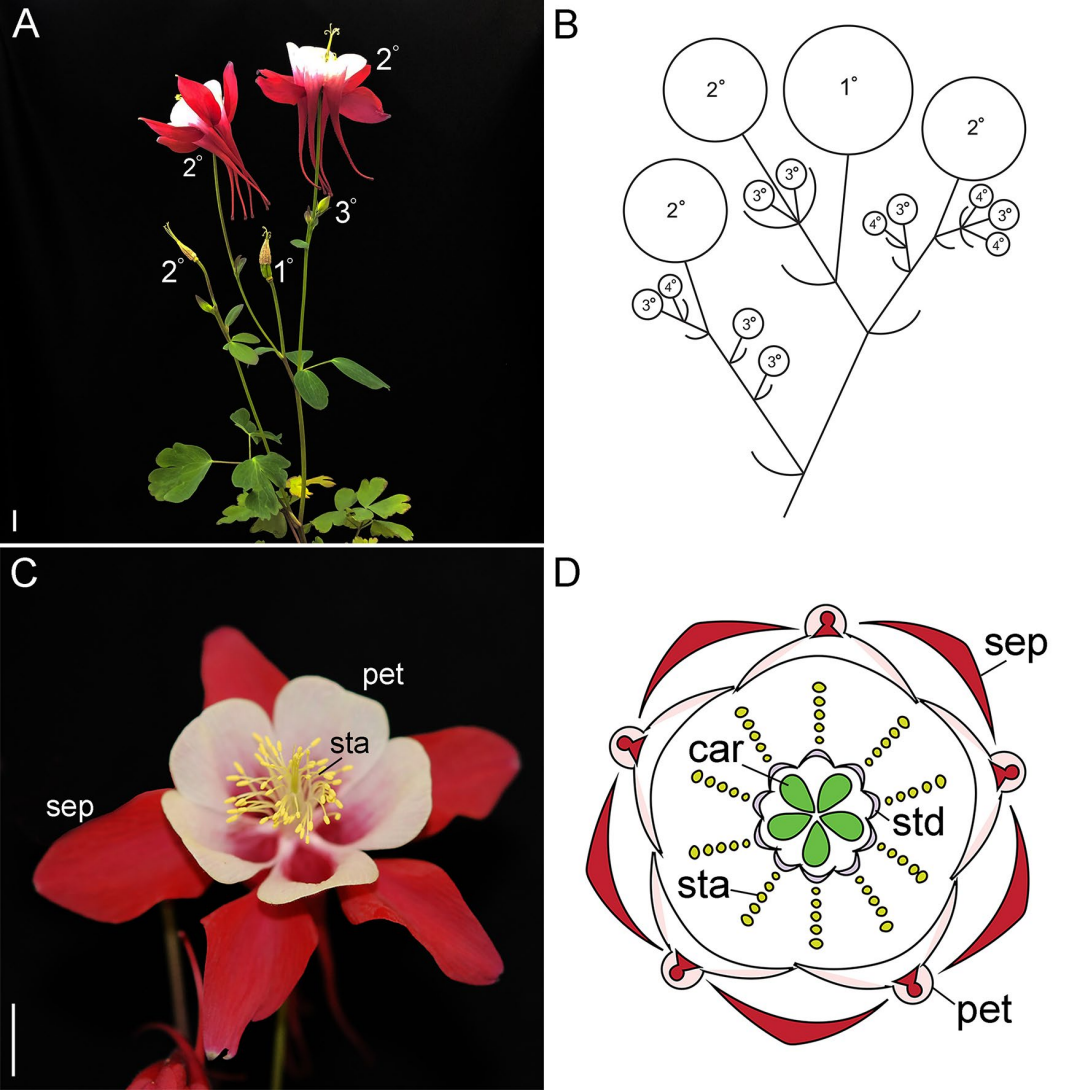
Wildtype reproductive morphology of *A. coerulea* ‘Origami.’ **(A)**. Reproductive phase plant with a late stage inflorescence. The flowers are labelled in order of maturity. The terminal flower, which has already shed its outer organs, is labeled 1º, followed by the subsequent flowers. The 3º and 4º flowers indicated in panel **(B)** are generally small buds in the axils of the subtending bracts, and therefore not visible at this scale. **(B)**. A schematic representing the primary inflorescence in panel **(A)**. **(C)**. A wildtype flower. **(D)**. A floral diagram. Sep, sepals; pet, petals; sta, stamens; std, staminodes; car, carpels. Size bars = 1 cm.

Homologs of all of these players have also been identified in models that produce cymose inflorescences such as *Petunia* and tomato, both members of the Solanaceae. In a cyme, inflorescence identity is transient rather than persistent (reviewed Bartlett and Thompson, 2014). While in the IM identity phase, the terminal meristem produces one to a small number of nodes before converting into a flower, which terminates growth on that axis. However, the existing axillary meristems can reiterate the sympodial pattern, with each axillary meristem expressing IM identity for some period before converting into a FM itself (e.g., **Figure 1B**). Detailed studies of the genetic basis of this pattern in *Petunia* and tomato reveal both similarities and differences relative to what has been found in racemes (reviewed Moyroud et al., 2010). As expected, *LFY* homologs are essential to the establishment of floral identity, but the spatial and temporal expression of FM identity is determined by the differential expression of *UFO* homologs, rather than the *LFY* homologs themselves (reviewed Moyroud et al., 2010). Thus, in *Petunia* and tomato, it would appear that much broader aspects of the LFY functional repertoire are dependent on UFO as a co-factor, although there is evidence that even in *A. thaliana*, *UFO* contributes towards floral meristem identity (Samach et al., 1999; Risseeuw et al., 2013). In these relatively closely related cymose models, IM identity does not strongly conform to what has been observed in *A. thaliana* and *A. majus*. Instead, it depends on other loci, including the WOX homolog *EVERGREEN* (*EVG*) and the ALOG family member *TERMINATING FLOWER* (*TMF*; Lippman et al., 2008; Rebocho et al., 2008; MacAlister et al., 2012).

Similar to *Petunia* and tomato, the lower eudicot model *Aquilegia* makes cymose inflorescences that are composed of monochasial or dichasial units (Kramer, 2009; **Figures 1A, B**). Following floral induction, which typically requires vernalization, the primary apical meristem converts into an IM (Ballerini and Kramer, 2011). This meristem produces two or more lateral bracts, which each subtends a new axillary IM (**Figure 1B**). The primary IM then transforms into a FM, which terminates the main axis. New growth of the primary inflorescence is taken over by the remaining axillary IMs, which reiterate the pattern. In our model system, *A. coerula* “Origami,” the primary inflorescence typically produces three to four bracts, while the subsequent axillary IMs of this inflorescence produce decreasing numbers of bracts (Kramer, 2009). The latest arising axillary meristems convert directly into FMs, thereby ceasing inflorescence branching. A similar pattern is observed in any secondary or tertiary inflorescences that arise from the basal rosette, but typically branching is reduced relative to the primary inflorescence. This pattern is clearly reflected in the expression of the previously characterized single *AqLFY* locus (Ballerini and Kramer, 2011). *AqLFY* is expressed on the flanks of the terminal meristem when it is in the inflorescence phase, but becomes strongly and constitutively expressed across the entire meristem once it transitions to floral identity (**Supplementary Figure S1**). This same pattern of expression changes is observed in each axillary meristem as it progresses from IM to FM identity. Expression then rapidly declines as the floral organs begin to initiate, persisting longest in the petals and carpels. Several putative inflorescence identity genes have also been identified in *Aquilegia*, including *AqAGL24.2*, which is strongly but transiently expressed in axillary meristems before they transition to FM identity, and *AqTFL*, which is expressed in a crescent-shaped wedge subtending early IMs (**Supplementary Figure S1**). The functional significance of these patterns remains to be explored, but they appear to underscore the importance of developmental timing in the generation of *Aquilegia*’s cymose inflorescence. The duration of the IM identity phase in any given meristem will determine how many bracts are produced, which in turn controls the branching and complexity of the inflorescence.

Once the meristem has acquired floral identity, it goes through a stereotypical developmental process of floral organ initiation (Tucker and Hodges, 2005). *Aquilegia* flowers have five distinct floral organ types: one whorl of five petaloid sepals, one whorl of five spurred petals, seven to ten whorls of five stamens each, two whorls of five staminodes each, and one whorl of five carpels (Kramer, 2009); **Figures 1C, D**). Investigation of the MADS box floral organ identity homologs has revealed a complex ABC model that incorporates three *APETALA3* (*AP3*) and two *AGAMOUS* (*AG*) homologs, as well as single copies of *PISTILLATA* (*PI*) and *FRUITFULL*-like (*FUL*), the closet relative to *AP1* (Kramer et al., 2007; Pabon-Mora et al., 2013; Sharma and Kramer, 2013; Zhang et al., 2013; Sharma and Kramer, 2017). The aspects of this work that are most relevant to our current study of *LFY* and *UFO* homologs are the findings regarding *AqFL1* and the three *AqAP3* paralogs. Unlike what has been observed for *euAP1* orthologs in the core eudicots (reviewed Litt and Kramer, 2010), *AqFL1* appears to promote the identity of IMs rather than FMs such that silencing of the gene results in decreased inflorescence complexity (Pabon-Mora et al., 2013). As for the *AP3* paralogs, they have experienced sub- and neofunctionalization such that *AqAP3-1* primarily promotes staminode identity, *AqAP3-2* controls stamen development, and *AqAP3-3* is specifically required for petal identity (Sharma et al., 2011; Sharma and Kramer, 2013).

These findings raise a number of questions regarding the *AquilegiaLFY* and *UFO* homologs. Given that there is no *AP1* ortholog and *AqFL1* appears to influence IM identity, do the *LFY* and *UFO* homologs play essential roles in FM identity? Are the distinct whorl-specific expression patterns of the *AP3* paralogs due to differential regulation by *LFY* or *UFO*? More broadly, how do the roles of *LFY* and *UFO* in the *Aquilegia* cyme compare to what has been observed in other cymose models such as *Petunia* and tomato? Our analysis of *AqLFY* and *AqUFO1/2* expression and function seeks to address these questions while highlighting still unknown aspects of the genetic control of inflorescence architecture in *Aquilegia*. Perhaps most significantly, neither *AqLFY* nor *AqUFO1/2* appear to be essential for FM identity, rather promoting a sharp transition from IM to FM identity. There may be evidence for a role for *AqUFO1/2* in specifically promoting *AqAP3-3* expression, but this is confounded by a potential parallel function in promoting the initiation of petals and outer stamens.

## Materials and Methods

### Plant Material

Seeds for *Aquilegia coerulea* “Origami Red and White” were obtained from Swallowtail Seeds (Santa Rosa, CA, USA), and germinated and grown under long day (16 hours light, 8 hours dark) at 18°C.

### Identification of *AqLFY* and *AqUFO* Homologs

*AqLFY* has been previously identified (Ballerini and Kramer, 2011) but was confirmed to be a single-copy locus in the most recent version of the *Aquilegia coerulea* (James) genome available on Phytozome v.12.1 (http://www.phytozome.net/). To identify *AquilegiaUFO* homologs, we conducted a BLAST search with default settings using the *Arabidopsis UFO* sequence as the query. Together with previously identified *UFO* orthologs and closely related F-box family members (Gagne et al., 2002), amino acid sequences were aligned using CLUSTALW (Larkin et al., 2007) as implemented in MacVector 15.5.4 (Gary, North Carolina) and then adjusted by hand to remove uninformative sequence. A maximum likelihood phylogenetic tree was constructed using RAxML (Randomized Axelerated Maximum Likelihood) v.8.0.0 {Stamatakis, 2014 #4210} with the default model of amino acid substitution as implemented on the CIPRES V.3.3 platform {Miller, 2009 #2920}. The resultant tree was displayed by FigTree v1.4.2 (http://tree.bio.ed.ac.uk/software/figtree/) and color prepared using Adobe Illustrator CC 2015. These analyses led to the assignment of gene names to each locus: *AqUFO1* (Aqcoe1G161900) and *AqUFO2* (Aqcoe4G199100). We should also note that there appears to be an almost identical copy of *AqUFO2* on an unassembled scaffold (Aqcoe0095s0001), but the predicted transcript differs by only a six nucleotide insertion in a repetitive stretch of the 3’ end of the coding region, making it extremely difficult to distinguish between them. For the purposes of this study, we are considering Aqcoe4G199100 and Aqcoe0095s0001 to be equivalent.

### *In Situ* Hybridization

Fragments of *AqUFO1* (383 bp) and *AqUFO2* (328 bp) from non-conserved regions of the open reading frame were PCR amplified using primers listed in **Supplementary Table S1**, and cloned into the pCR™4-TOPO^®^ vector. Both sense and anti-sense probes of each gene were alkaline hydrolyzed to an average length of 150 bp. All *in situ* hybridization steps were performed as described by Kramer (2005). Slides were visualized on the Zeiss AxioImager microscope at the Arnold Arboretum of Harvard University.

### Virus Induced Gene Silencing

The *Aquilegia* VIGS protocol and construction of the TRV2-*AqANS* positive control plasmid has been described previously (Gould and Kramer, 2007; Kramer et al., 2007; Sharma and Kramer, 2013). To make the TRV2-*AqLFY*-*AqANS* and TRV2-*AqUFO2*-*AqANS* constructs, we PCR amplified a 532 bp fragment of *AqLFY* and a 389 bp fragment of *AqUFO2* using primers that added BamHI and KpnI sites to the respective 5′ and 3′ end of the PCR products (see the **Supplementary Table S1**). Similarly, *AqUFO1* constructs were prepared with primers that added EcoRI and Xba sites to a 359bp fragment (**Table S1**). The TRV2–*AqUFO1*–*AqUFO2*–*AqANS* construct was made using the same fragments as in the individual constructs. The regions used to prepare the VIGS constructs share 71% identity. For each treatment, including the control *TRV2–AqANS*, 100-150 *Aquilegia coerulea* “Origami Red and White” plants at the ∼5 leaf stage were vernalized at 4°C for 3 weeks and then treated as described in Gould & Kramer (2007) as soon as they were removed from cold treatment. After multiple failed silencing attempts using the standard protocol, we found that this short vernalization treatment was necessary to achieve the early inflorescence silencing needed to affect *AqLFY* and *AqUFO1/2*. Following the treatment, plants were returned to the long day conditions at 18˚C. Flowers showing any *AqANS* silencing were photo-documented and, upon maturation, the flowers were dissected and organs counted. The organ count datasets were subjected to ANOVA to detect differences between treatment classes followed by Scheffé’s test to detect pairwise difference between the classes, which have unequal sizes. All individual organs were photographed using a Kontron Elektronik ProgRes 3012 digital camera mounted on a Leica WILD M10 dissecting microscope (Harvard Imaging Center; Cambridge, MA, USA). For every flower showing silencing, a selection of organs from each whorl was frozen at -80°C for subsequent RNA analysis.

### Expression Analysis of VIGS-Treated Organs

Total RNAs were prepared from collected floral buds and organs using the Qiagen RNeasy kit according to manufacturer’s instructions (Qiagen, Valencia, CA, USA). Each RNA sample was treated with TURBO DNase (Ambion by Life Technologies, Carlsbad, CA, USA) following manufacturer’s instructions. Individual RNAs were quantified using Nanodrop (ThermoFisher Scientific, Waltham, MA, USA) and cDNAs were synthesized using 1 µg of total RNA (SuperScript III™ First Strand Synthesis, Invitrogen, San Diego, CA, USA). All primer sequences are listed in **Supplementary Table S1**. The quantitative RT-PCR was carried out using PerfeCTa qPCR FastMix, Low ROX (Quanta Biosciences Inc., Gaithersburg, MD, USA) in the Stratagene Mx3005P QPCR system (Agilent, Santa Clara, CA, USA). The PCR program was: 10 minutes at 95°C; followed by 40 cycles of 30sec at 95°C, 30sec at 56°C, 30sec at 72°C; and, finally, 1 cycle of 30sec at 55°C, 30sec at 95°C. At least one of the qRT primer pairs for each gene was designed to span an intron position. *AqIPP2* (*ISOPENTYL PYROPHOSPHATE : DIMETHYLALLYL PYROPHOSPHATE ISOMERASE2*; GenBank KC854337) was used for normalization as it has been previously shown to have little quantitative transcriptional variation across tissues and developmental time points (Sharma et al., 2011). Primer efficiencies were evaluated using six 1:4 dilution series, and all showed efficiencies above 90%. Three to five biological replicates for both control and VIGS treated tissue were examined. Expression for each biological sample was assayed from three replicates per reaction plate. The variability resulting from technical replicates was negligible compared to the variability from biological replicates, so only biological variability is presented here. Relative gene expression levels were calculated using the 2^-ΔΔC^_T_ method described in Livak and Schmittgen (2001), taking into consideration the specific primer efficiencies as well as the exact fragment lengths.

To assay down-regulation of *AqLFY*, *AqUFO1* and *AqUFO2*, we dissected six to eight stage 8-10 floral buds exhibiting multiple sepal whorls from silenced inflorescences. RNA was prepared from each meristem and tested as a separate bioreplicate. This was to address the fact that these loci are not expressed in mature floral organs. Comparable buds were dissected from *AqANS*-silenced inflorescences to serve as controls. The dissected mature organs from silenced flowers were assessed for expression of *AqAP3*-*1, AqAP3*-*2*,*AqAP3-3*, and *AqPI*. For each class of dissected organs, we analyzed three to five separate bioreplicates, and each bioreplicate was analyzed in three technical replicates. A two-tail Mann Whitney U Test was used to determine the statistical significance of differences between experimental and control values from *AqANS*-silenced plants. All results presented are the mean ± standard deviation of the examined samples, normalized to the appropriate reference samples as described above. All error bars represent standard deviation

### Yeast Two-Hybrid Study

The entire coding regions of *AqLFY*, *AqUFO1*, and *AqUFO2* were PCR-modified with primers containing 5’ and/or 3’ flanking EcoRI sites and cloned into pCR4-TOPO (Invitrogen). The subsequent inserts were cloned in-frame into pGBKT7 or pGADT7 vectors (Clontech Mountain View, CA). Indicated Y2H protein pair combinations (**Table 1**, **Supplementary Figure S4**) were sequentially transformed into yeast host strain AH109. Manufacturer’s positive (p53/T-antigen) control was used to monitor Y2H assays. Two *Aquilegia vulgaris* MADS proteins (AqAP3-1, AqPI), previously shown to have strong protein–protein interactions in this system (Kramer et al., 2007), were also used as an additional Y2H positive control. For more details of Y2H protocol see Kramer et al. (2007) or Holappa et al. (2017).

**Table 1:** Yeast two-hybrid results.

Constructs	AqLFY-AD	AqUFO1-AD	AqUFO2-AD
**AqLFY-BD**	+	++	++
**AqUFO1-BD**	++	++	+
**AqUFO2-BD**	-	-	-

## Results

### *AqLFY*-Silenced Phenotypes and Effects on Gene Expression

**Figure 2:**
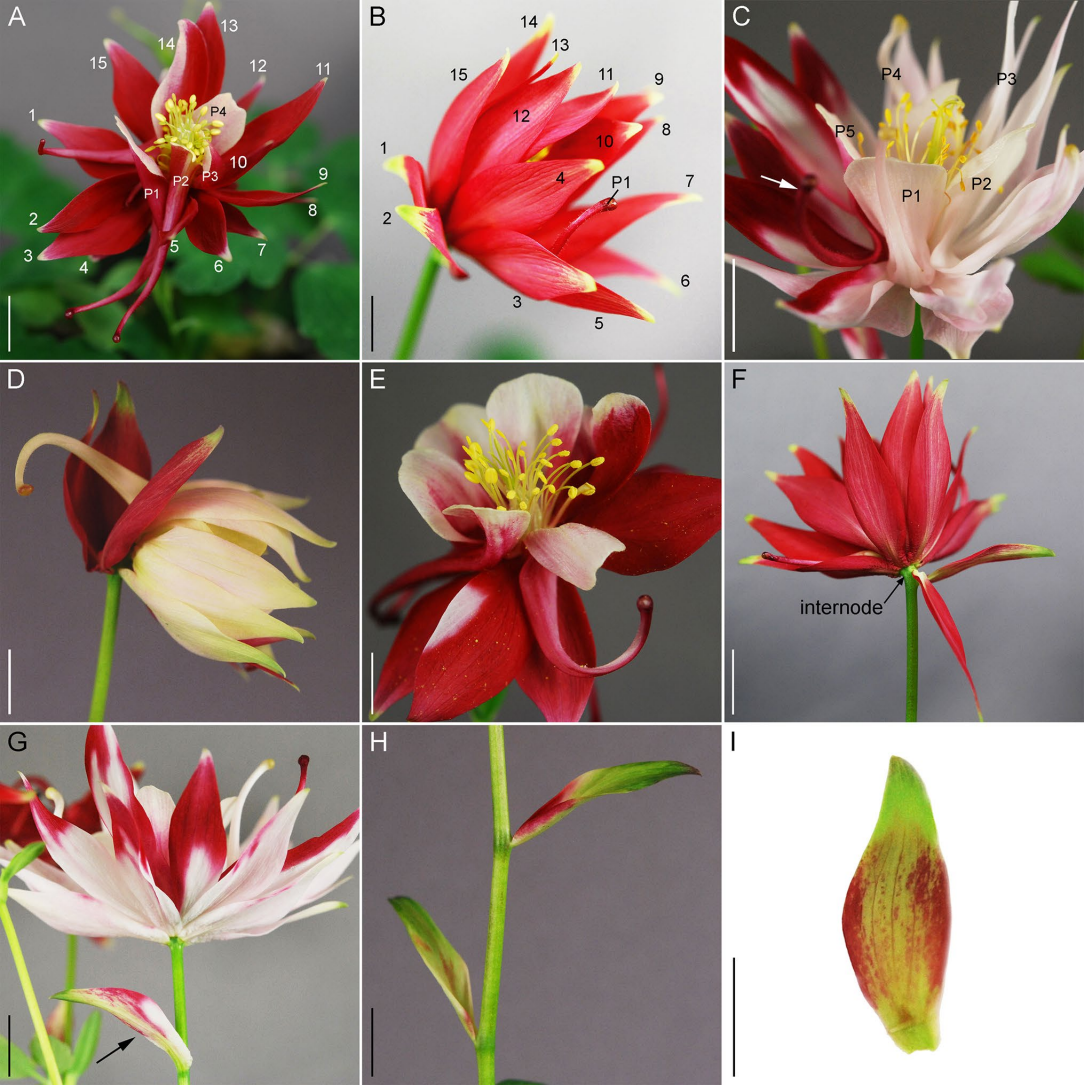
*AqLFY*-silencing phenotypes. **(A)** Flower with two additional whorls of sepals (numbered) and four petals (P1-4). **(B)** Side view of flower with two additional whorls of sepals. Most petals are not visible, with the exception of P1. **(C)** Flower with additional sepals, only petals numbered (P1-5). Note how additional sepals cause the petal spurs to bend upward (white arrow). **(D)** Side view of flower with additional sepals, one petal visible. **(E)** Front view of flower with additional sepals, note normal morphology of internal floral organs. **(F)** Back view of flower with additional sepals, note short internode between outermost sepals and first continuous whorl of organs. **(G)** Side view of flower with additional sepals and a bract/sepal chimera (black arrow). **(H)** Bract/sepal chimeras that subtend no active axillary meristems. **(I)** Individual bract/sepal chimera. Size bars = 1 cm.

**Figure 3:**
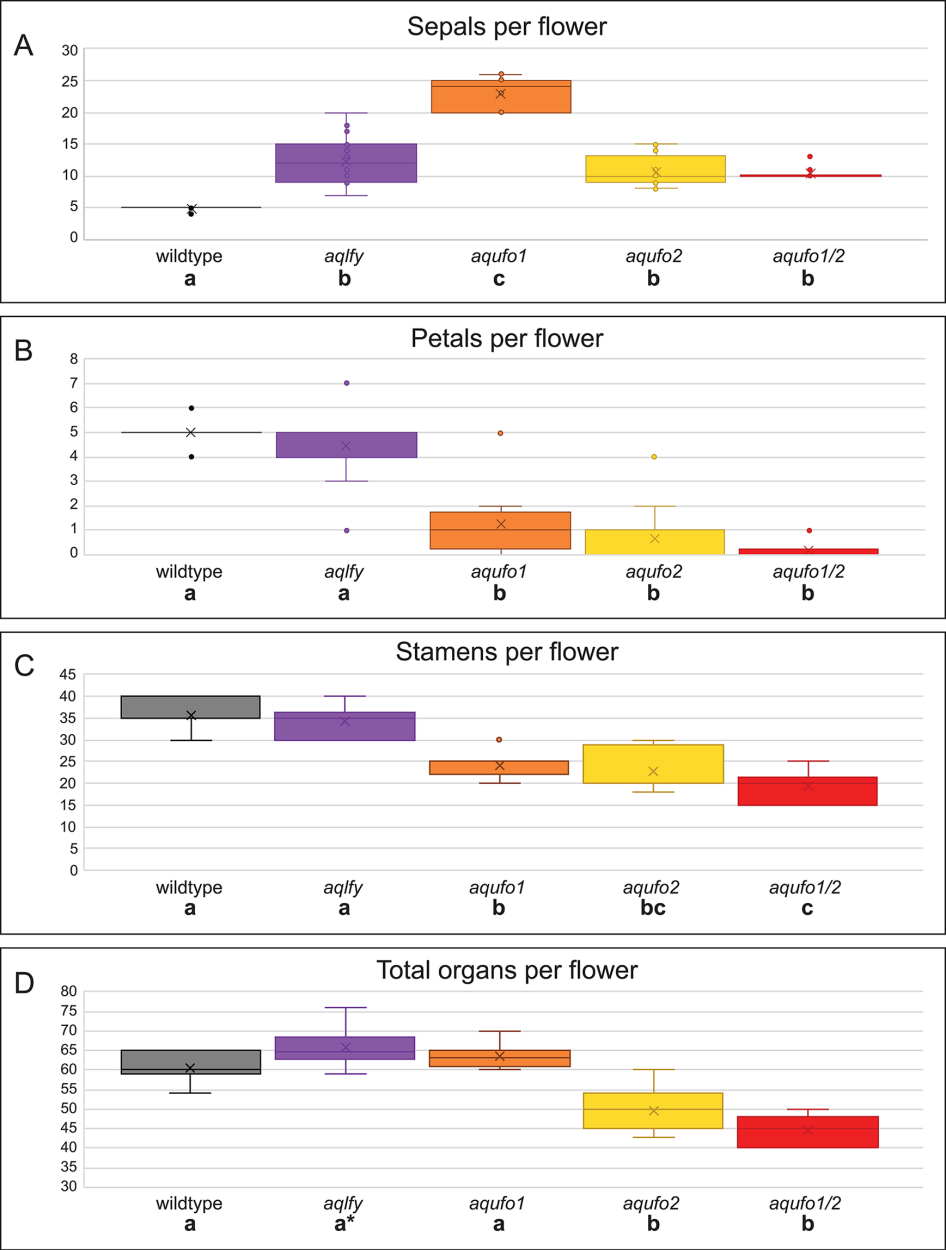
Organ counts for wildtype and silenced flowers. Flowers were dissected from wildtype (*AqANS*-silenced control, n = 15), *aqlfy* (n = 38), *aqufo1* (n = 71), *aqufo2* (n = 32), and *aqufo1/2* (n = 25) cohorts. The per flower distributions above are presented for sepals **(A)**, petals **(B)**, stamens **(C)**, and total organs **(D)**, while the staminode and carpel values, which did not differ across cohorts, are presented in **Supplemental Figure S2**. For each class of data **(A**-**D)**, a one-way ANOVA was conducted to determine whether any of the means were statistically different, followed by a Scheffé test to determine which means differed from one another. The statistically different classes are indicated below the cohort labels by lettered labels (a-c), where the same letter indicates no difference between the classes and different letters indicate a difference at p < 0.01. In panel **(C)**, note that the *aqufo2* could not be differentiated from *aqufo1* or *aqufo1/2*, although the two latter classes could be differentiated from each other. In panel **(D)**, the asterisk for *aqlfy* reflects the fact that this class was differentiated from the wildtype at p < 0.05, but not the p < 0.01 standard used for the remainder of the comparisons.

We treated 100 A*. coerulea* “Origami” plants with *Agrobacterium* containing TRV1 and TRV2-*AqLFY-AqANS*. 24 plants produced 38 flowers showing distinct silencing phenotypes, which we will term *aqlfy* (**Figures 2A–G**). As a point of reference, dicot *lfy* mutants characterized to date typically show partial to complete loss of FM identity, and when flowers are produced, they show conversion of floral organs towards leaf identity, particularly of the outer whorls, as well as shifts towards spiral phyllotaxy (reviewed Moyroud et al., 2010). In contrast, *aqlfy* plants produced many flowers and their most notable phenotype was the presence of two to three whorls of five sepals each, which are produced before the flower progresses to making morphologically normal petals, stamens, staminodes and carpels. The average number of sepals per flower were significantly higher (p < 0.01) than the *AqANS*-silenced controls, although there were no significant differences in the numbers of each of the other floral organs (**Figure 3**, **Supplementary Figure S2**). The total number of organs per flower were not significantly different at p < 0.01, but this comparison is complicated by the high variation in stamen number per flower, which ranges from 30 to 45 between flowers in an inflorescence. The total number of organs per flower were statistically higher in *aqlfy* at p < 0.05. The additional organs were arranged in alternate whorls relative to the outermost whorl. Although the presence of the extra sepal whorls did cause the petal spurs to reflex backward (**Figure 2C**), the overall morphology of the floral organs was not significantly affected and chimeric floral organs were relatively rare. However, the petioles of silenced flowers often bore bract/sepal chimeras that were separated by internodes (**Figures 2B–I**). Like sepals, these organs were lanceolate in shape, had red and white sectors, lacked the sheathing base typical of *Aquilegia* bracts, and subtended no obvious axillary meristems. The internodes separating these organs were variable in length and their phyllotaxy was typically spiral (**Figure 2H**).

**Figure 4:**
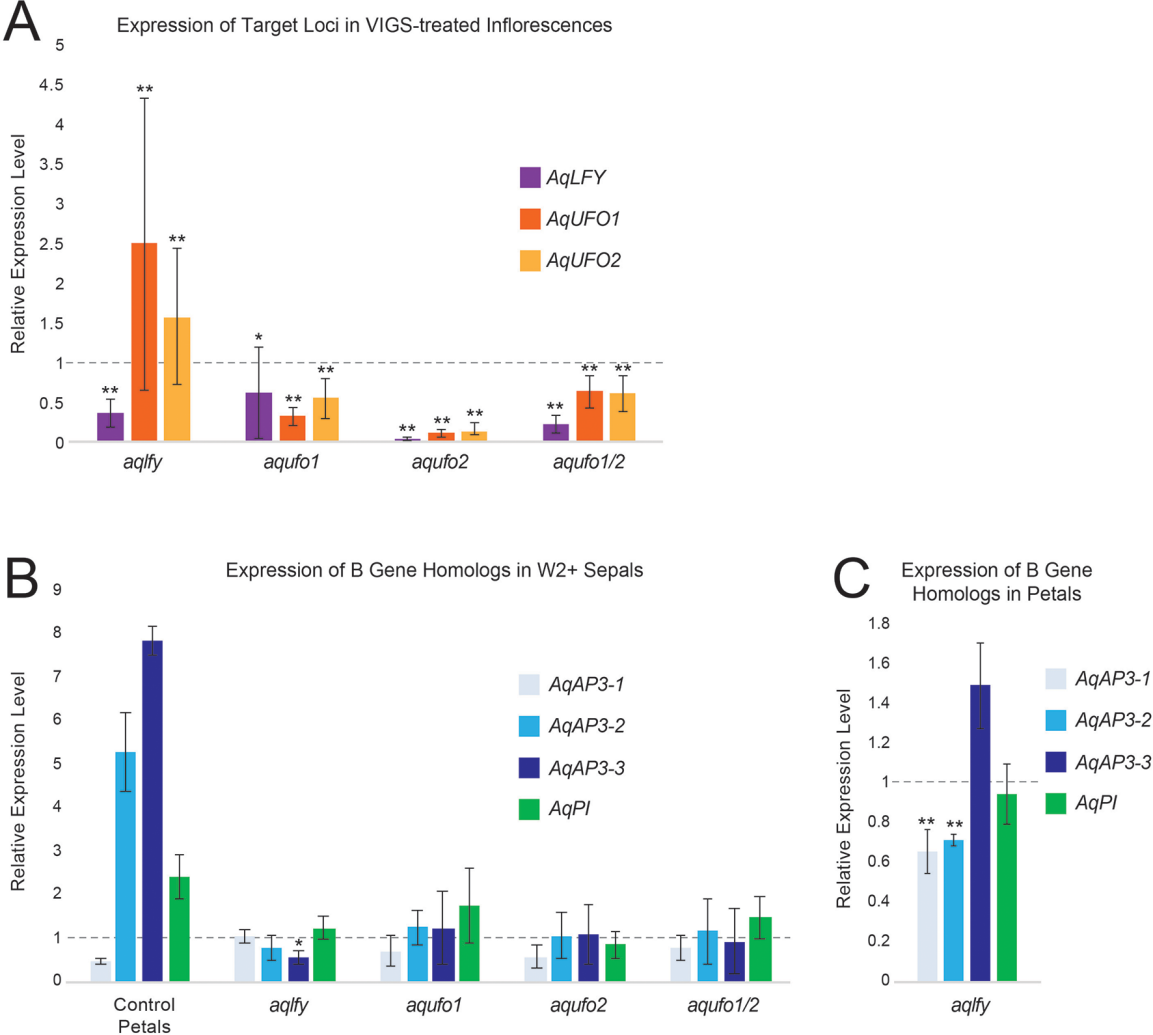
RT-qPCR results for *AqLFY-*, *AqUFO1*-, *AqUFO2*-, and *AqUFO1/2*-silenced material. **(A)**. Expression of *AqLFY, AqUFO1*, and *AqUFO2* in six to eight separate bioreplicates of putatively silenced stage 8-10 floral meristems. All values are normalized to the expression of each locus in control *AqANS*-VIGS meristems of comparable stage (dotted line). **(B)**. Expression of the three *Aquilegia AP3* homologs (*AqAP3-1*, *-2*, and *-3*) and the single *AqPI* homolog in the inner sepals produced in each class of silenced flower. For reference, we show the expression levels of these loci in the control second whorl petals of *AqANS*-VIGS flowers. All values are normalized to the expression levels of each gene in control sepals (dotted line). **(C)**. Expression of the three *Aquilegia AP3* homologs (*AqAP3-1*, *-2*, and *-3*) and the single *AqPI* homolog in the second whorl petals produced in *AqLFY*-silenced flowers. All values are normalized relative to the expression of each gene in control petals (dotted line). All vertical axes represent relative expression levels, all asterisks indicate expression levels that are significantly different from the reference control organs based on the two-tail Mann Whitney U Test, and all error bars represent standard deviation (*p<0.01, **p<0.05).

Confirming that *AqLFY* was down-regulated in these flowers was complicated by the fact that the gene is not expressed at detectable levels in mature floral organs (Ballerini and Kramer, 2011). The approach we took was to dissect the earliest possible axillary flower buds (stages 8-10, Ballerini and Kramer, 2011) from silenced inflorescences and then confirm that they had multiple whorls of sepals. This allowed us to confim that *AqLFY* is down-regulated in floral buds of the same inflorescences that show abnormal phenotypes. Expression levels in each of these buds were compared to those in equivalent staged buds from control *AqANS*-silenced plants. This approach did allow us to confirm that *AqLFY* is downregulated in floral buds showing mutant phenotypes (**Figure 4A**). We also assessed expression of *AqUFO1* and *2* (see below) in these *aqlfy* buds and found that both loci appeared to be up-regulated, especially *AqUFO1*. The B gene homolog expression levels were generally similar to control sepals in the additional sepal whorls, as well as in the petals (**Figures 4B, C**), with the exception of *AqAP3-1* and *AqAP3-2* expression, which was somewhat reduced in the petals.

### Identification of *Aquilegia UFO* Homologs and Their Expression Patterns

**Figure 5:**
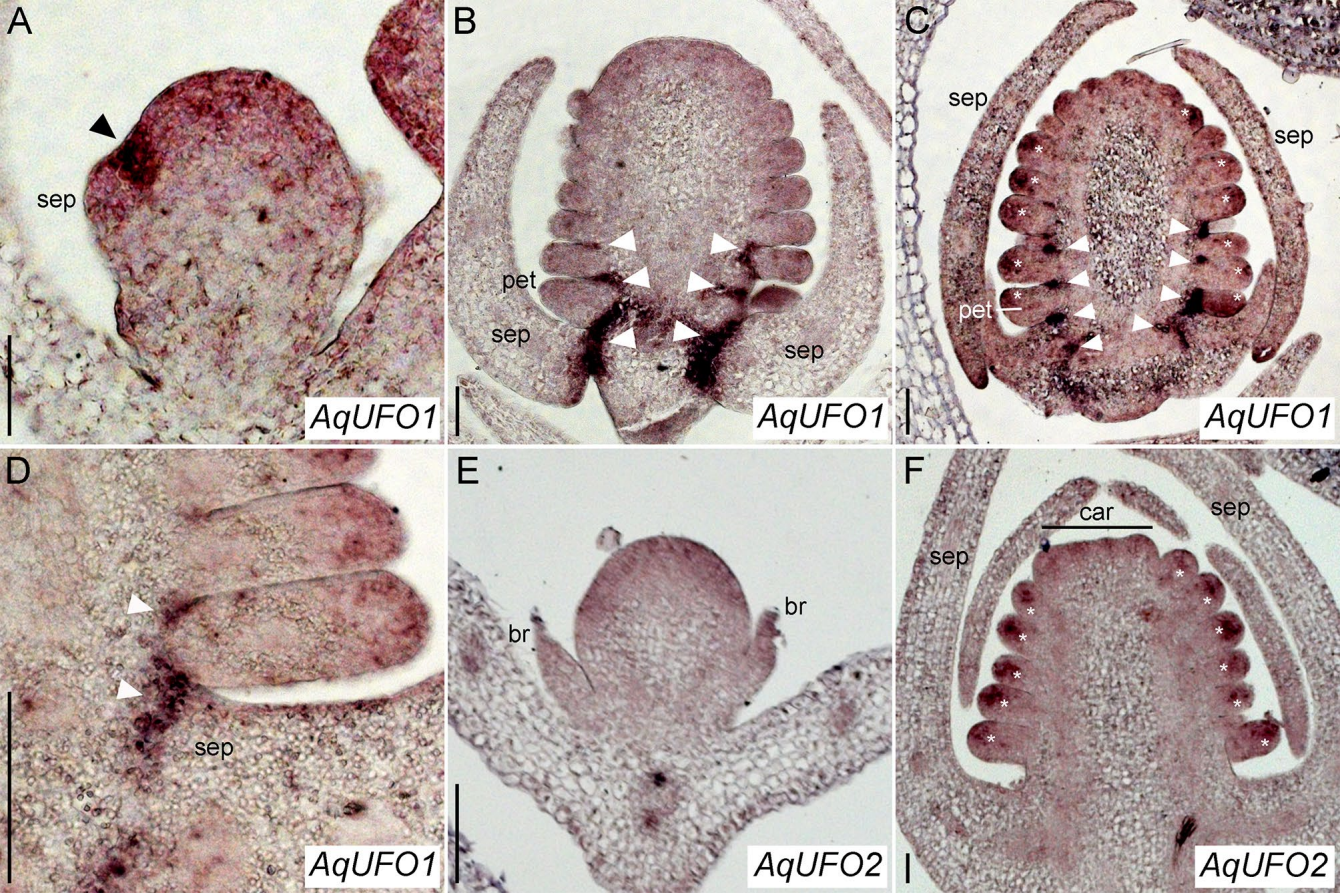
*AqUFO1* and *2 in situ* hybridization patterns. **(A**–**D)** *AqUFO1* expression. **(E**–**F)**. *AqUFO2* expression. **(A)** Early stage 3 floral meristem showing strong *AqUFO1* expression (arrowhead) in the axil of an arising sepal (sep) primordium. **(B)** Mid-stage 6 floral meristem in which *AqUFO1* expression (arrowheads) has expanded into the bases of the sepals (sep), petals (pet, as judged by wedge-shape), and outer stamen primordia. **(C)** Mid/late stage 6 floral meristem showing *AqUFO1* expression (arrowheads) persisting in the bases of the sepals (sep), petals (pet), and outer two whorls of stamens. In addition, expression is detected at the distal ends of the petal and stamen primordia (asterisks). Note, this section is slightly tangential so most of these primordia are likely stamens, although the basalmost primordium on the left, which is larger, wedge-shaped and upward turning at the tip, is likely a petal. **(D)** Close up of *AqUFO1* expression (arrowheads) in an early stage 7 floral meristem. It is unclear whether the first primordium after the sepal is a petal or a stamen. **(E)** *AqUFO2* expression in a stage 2 floral meristem subtended by two bracts (br). Expression appears diffuse and moderate. **(F)** *AqUFO2* expression in an early stage 7 floral meristem. Signal is only detected at the distal ends of the floral organ primordia (asterisks). Early carpel (car) primordia are visible at the apex but it is unclear whether the basalmost primordia after the sepals (sep) are stamens or petals. Size bars = 50 µm.

There are 485 annotated members of the F-box gene family in the *Aquilegia* genome (Filiault et al., 2018). We identified representatives of the C5 subfamily known to contain *UFO* (Gagne et al., 2002) using BLAST and conducted a phylogenetic analysis (**Supplementary Figure S3**), which revealed two recent paralogs, termed *AqUFO1* and *AqUFO2*, as the closest homologs of previously characterized *UFO*-like genes from other model systems. Expression studies of *AqUFO1* and *2* in other tissues have not detected appreciable levels of *AqUFO1* or *2* expression outside the inflorescence (data not shown), so we used *in situ* hybridization to determine the expression of these genes in developing floral meristems (**Figure 5**). *AqUFO1* is expressed at diffuse, moderate levels in early stage floral meristems but expression increases dramatically in the axils of the sepals as they initiate (**Figure 5A**). As subsequent floral organs arise, high expression is detected in the bases of the developing sepals, petals, and outer stamen whorls (**Figures 5B–D**). In addition, as the stamens begin to differentiate, moderate expression appears to become concentrated in the distal tips of the organs (**Figure 5C**). The expression of *AqUFO2* is seen as broad and moderate at the earliest stages of floral meristem development, and in the distal tips of developing stamens at later stages, but no expression is detected at the bases of the sepals, petals, or stamens (**Figures 5E, F**).

### *AqUFO1/2*-Silenced Phenotypes and Effects on Gene Expression

**Figure 6:**
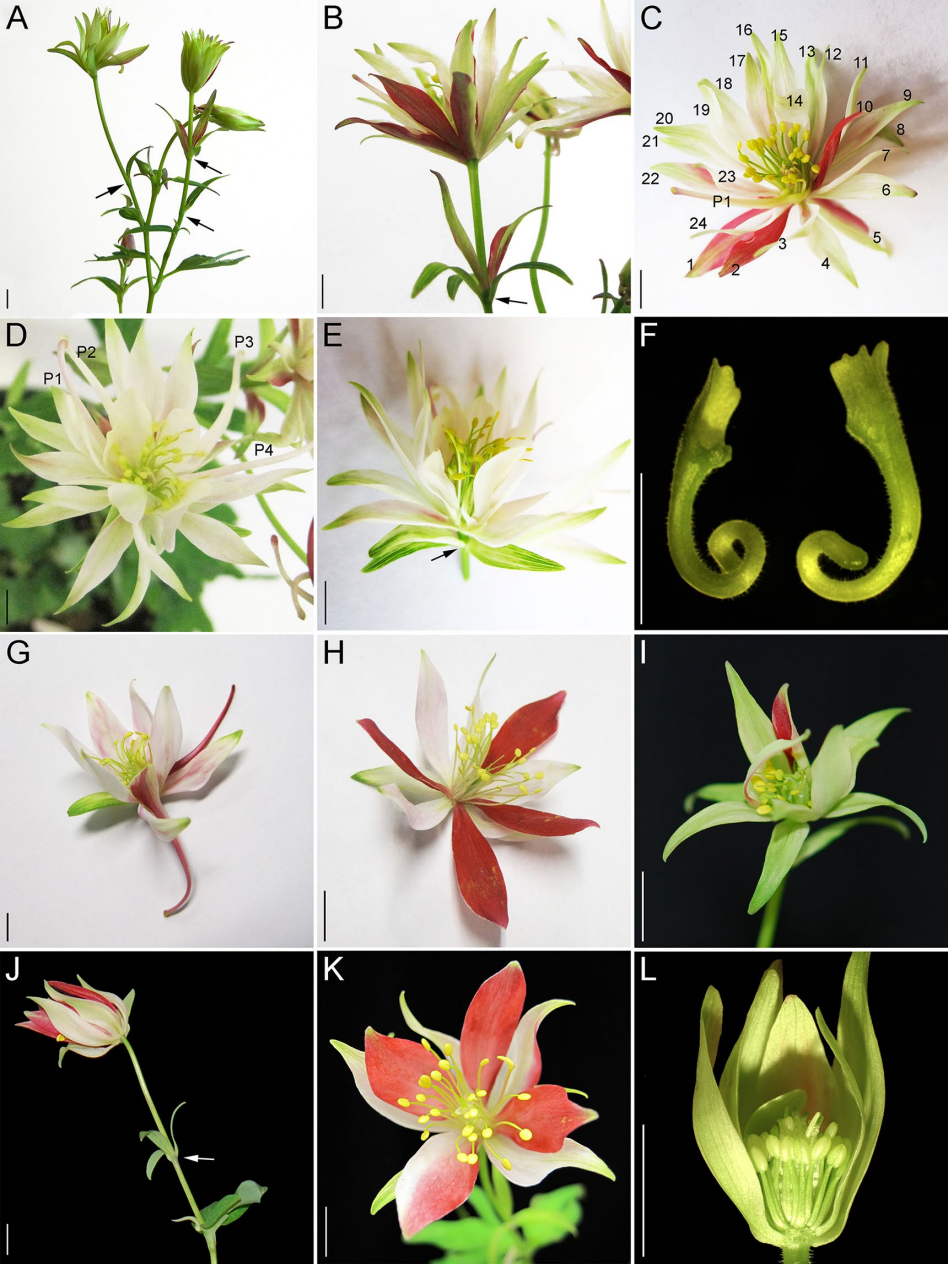
*AqUFO1*- and *2* single and double silencing phenotypes. **(A**–**F)** *AqUFO1*-silenced inflorescences, flowers and floral organs. G-H. *AqUFO2*-silenced inflorescences and flowers. I-L. *AqUFO1/2*-silenced flowers. **(A)** Inflorescence with multiple bract/sepal chimeras that subtend no active axillary meristems (black arrows). **(B)** Flower with extra whorls of sepals and a petiole that bears an entire whorl of bract/sepal chimeras (black arrow). **(C)** Flower with extra whorls of sepals (numbered) and one petal (P1). **(D)** Flower with extra whorls of sepals and four petal (P1–P4). **(E)** Flower with extra whorls of sepals and a petiole that bears a whorl of bract/sepal chimeras (black arrow). **(F)** Two stunted petals with reduced blades. **(G**–**H)**. *AqUFO2*-silenced flowers with two complete whorls of sepals and either two **(G)** or no **(H)** petals. **(I)** *AqUFO1/2*-silenced flower with two whorls of sepals and reduced stamen numbers. **(J)** *AqUFO1/2*-silenced inflorescence bearing multiple bracts with no active axillary meristems. **(K**–**L)** Flowers with two whorls of sepals, no petals, and reduced stamen numbers. Three sepals and two stamens were removed to show the inner whorls in L. Size bars = 1 cm.

We treated 100-150 plants each with *Agrobacterium* containing TRV1 and TRV2-*AqUFO1-AqANS*, TRV2-*AqUFO2-AqANS* or TRV2-*AqUFO1-AqUFO1-AqANS*. In each treatment class, 23-30 plants produced 25-70 flowers showing distinct silencing phenotypes, which we have respectively termed *aqufo1* (29 plants, 71 flowers), *aqufo2* (23 plants, 32 flowers), and *aqufo1/2* (20 plants, 25 flowers; **Figure 6**). Again, for comparison, the phenotypes of dicot *ufo* mutants generally fall into two classes: 1) in *Petunia* or tomato, strong loss of FM identity (very similar to strong *lfy*) or 2) in *A. thaliana* or *A. majus*, transformation of petals and stamens towards sepals or filaments, respectively, as well as variation in organ number and disorganized phyllotaxy (reviewed Souer et al., 2008). In general, the observed *aqufo* floral phenotypes were similar to those in *aqlfy* plants, with some notable differences. For *aqufo1*, flowers had extra sepal whorls but also a possible transformation of petals into sepals (**Figures 6**A–E). Thus, the number of sepals per flower was even higher in *aqufo1* plants than in *aqlfy*, while the number of petals was much lower, typically zero to two (**Figure 3**; **Supplementary Figure S2**). Petals sometimes appeared stunted with incompletely developed blades (**Figure 6F**). The other notable difference in these flowers was a decreased number of stamens, which were present in only three to five whorls rather than the six to ten of control flowers (**Figure 3**). Also similar to the *aqlfy* phenotype, the inflorescences of the *aqufo1* plants bore bract/sepal chimeras (**Figures 6A, B, E**), which could be solitary (**Figure 6A**) or organized in whorls that were separated from the actual flower by variably elongated internodes (**Figures 6B, E**).

The *aqufo2* and *aqufo1/2* phenotypes were essentially identical to each other, but slightly distinct from *aqufo1*. These flowers exhibited fewer extra sepal whorls such that there were only two whorls of sepals and few to no petals (**Figures 3** and **6G–L**). Again, stamen numbers were consistently reduced but staminodes and carpels were unaffected (**Figure 3**; **Supplementary Figure S2**). Sepal/bract chimeras were still observed (**Figure 6J**).

As described above for *AqLFY*, we used stage 8-10 buds from silenced inflorescences to confirm the silencing of *AqUFO1* and *AqUFO2*. Expression levels in each of these buds were compared to those in equivalent staged buds from control *AqANS*-silenced plants. This approach did allow us to confirm silencing in flowers showing mutant phenotypes (**Figure 4A**), but notably, this response did not appear to be locus specific. Both *AqUFO1* and *AqUFO2* show down-regulation in all three treatment cohorts. In addition, *AqLFY*, also shows reduced expression in the various *AqUFO*-silenced meristems. The B gene homolog expression levels of the additional sepal whorls were generally similar to control sepals, consistent with their morphology.

### Protein–Protein Interactions

We used yeast two-hybrid to test the ability of AqLFY to interact with *AqUFO1* and *AqUFO2*. These assays detected weak to moderate interactions between *AqLFY* and both UFO paralogs (**Table 1**, **Supplementary Figure S4**), although the *AqLFY/AqUFO2* interaction was only recovered in one direction (AqLFY-BD/AqUFO2-AD).

## Discussion

### The Roles of *AqLFY* and *AqUFO1/2* in Inflorescence Architecture

The architecture of cymose inflorescences is fundamentally about timing (reviewed Bartlett and Thompson, 2014; Park et al., 2014)—How long is the indeterminate IM identity program expressed in a given meristem before it transitions to determinate FM identity? In the previously studied cymose models of the Solanaceae, this timing is primarily controlled by differential expression of *UFO* homologs (Park et al., 2014). *LFY* homologs are fairly broadly expressed but the expression of *UFO* homologs is restricted to the flowers, and it is *UFO* up-regulation that is critical for the IM to FM identity transition. In contrast, in several racemose models, *LFY* expression is more specific to floral meristems and it is *UFO* that is more broadly expressed, including in vegetative meristems (reviewed Moyroud et al., 2010). In *Aquilegia*, the *AqLFY* and *AqUFO1/2* expression patterns appear to fit relatively well with the cymose model: *AqLFY* is expressed in vegetative lateral organs but increases in the flanks of IMs and then becomes constitutive in FMs (Ballerini and Kramer, 2011), while *AqUFO1* and *2* appear to be more specific to FMs (**Figure 5**; data not shown). In detail, *AqUFO1* expression is relatively similar to what is observed in core eudicot *UFO* orthologs (Kusters et al., 2015), with some differences that may reflect the number of floral whorls in *Aquilegia*. All of the previously examined model systems have only four whorls of floral organs and *UFO* ortholog expression is primarily found at the sepal/petal interface (Kusters et al., 2015). In *Aquilegia*, *AqUFO1* is expressed in this domain but is also seen in the interface zones of the subsequent two whorls of stamens. It remains to be seen whether the pattern observed in *Aquilegia* is common in taxa with multiple stamen whorls, or is unique to this lineage. In contrast, *AqUFO2* does not exhibit the boundary expression domains but appears concentrated in the distal region of floral primordia. This represents an expression pattern that has not been widely observed in *UFO* orthologs. *ABERRANT PANICLE ORGANIZATION* (*APO*), the rice *UFO* ortholog, is quite broadly expressed, including in lateral organs, but its function appears to be largely unrelated, as well as novel, in that it delays the transition to FM identity rather than promoting it (Ikeda et al., 2005; Ikeda et al., 2007).

So based on expression, we might expect to see phenotypes similar to what has been observed in *Petunia*: strong loss of floral meristem identity in both *aqlfy* and *aqufo* silenced plants (reviewed Moyroud et al., 2010). Instead, the primary phenotype we observed is a stepwise delay in the IM to FM transition. In *Aquilegia*, the IM identity program results in the production of leaf-like bracts that subtend new axillary IMs and have moderately elongated internodes, while the FM identity program generates floral organs that have no axillary meristems and highly compressed internodes. In all of our silenced cohorts, it appears that the IM to FM transition occurs in a gradual manner such that one or more nodes of bract/sepal chimeras are produced before the final commitment to FM identity. Even after the initiation of what we would term a flower, as indicated by whorled phyllotaxy and compressed internodes, floral development does not proceed normally, resulting in multiple whorls of sepals.

Interpreting these phenotypes is complicated by the fact that the expression of *AqLFY* and both *AqUFO1* and *2* rapidly declines during stages 8 and 9 as the floral organs begin to differentiate, meaning that expression cannot be directly assayed in mature silenced flowers. Our approach of testing the youngest possible floral buds that showed altered morphology does appear to have confirmed that each of the target loci are substantially silenced in their respective cohorts, but the data is not without difficulty. When *AqLFY* is targeted, it appears that *AqUFO1* and *2* expression increases, particularly *AqUFO1* (**Figure 4A**). We believe that this is due to the fact that *AqUFO1* is primarily expressed at the boundary of the outer floral whorls. Silencing of *AqLFY* results in additional sepal whorls, which in turn would increase the amount of *AqUFO1*-expressing tissue. In the *aqufo1*, *aqufo2* and *aqufo1/2* plants, there are two issues. First, regardless of whether we targeted only one or both paralogs, both copies appear to be down-regulated. This could be the result of off-target silencing between the relatively similar paralogs, or due to transcriptional cross-regulation. Second, we see reduced *AqLFY* expression in all of these cohorts. This is most likely an indirect effect of the loss of petals in these flowers. *AqLFY* is expressed the longest in petals and carpels, so the absence of petals would be likely to lower *AqLFY* expression. That being said, we cannot rule out the possibility that *AqUFO1/2* silencing feeds back onto *AqLFY* expression due to an impact on FM identity.

Setting aside these caveats, it remains the case that none of our silenced phenotypes resemble the strong loss of FM identity that is observed in most core eudicot *LFY* or cymose *UFO* mutants. Notably, the *Aquilegia* phenotypes are reminiscent of what Wreath et al. observed when they silenced *EcFLO*, a *LFY* ortholog in *Eschscholzia californica* (Wreath et al., 2013). *Eschscholzia* is a member of the Papaveraceae, which is deeply diverged from *Aquilegia*’s family, the Ranunculaceae, within the order Ranunculales (Becker et al., 2005). Wreath et al. (2013) observed the repeated production of sepal whorls, including some instances of internodal elongation between the early whorls, before the meristems finally transitioned to the production of the other floral organs. This is closely analogous to our phenotype of sepal-like organs arising with variable phyllotaxy and degrees of internodal elongation.

In the flower itself, one possible explanation for the additional sepal whorls is that they are connected to the initiation of B gene expression. Both *LFY* and *UFO* typically play roles in activating the B class genes, particularly *AP3*, which then participates with the other B gene *PISTILLATA* (*PI*) in an auto-regulatory feedback loop that stabilizes their mutual expression (reviewed Smyth, 2018). Consistent with this, it is common to see classic “B class” homeosis in both *LFY* and *UFO* mutants (reviewed Moyroud et al., 2010), resulting in the transformation of petals into sepals and stamens into carpels. However, we did not see any homeosis of this type in *AqLFY* and we saw no evidence of stamen to carpel homeosis in any of the cohorts (see below for a consideration of the petal loss in *AqUFO*). In *Eschscholzia*, the authors did see sepal/petal chimeras, but these appeared to represent a transformation grade from outer sepals to inner organs that had full petal identity. Therefore, while these phenotypes are consistent with a delay in activating B gene expression, once transcription is initiated, there is no evidence of a significant problem with *AP3/PI* expression.

An alternative explanation that could explain both the sepal/bract chimeras and the additional sepal whorls in the flower is a failure to definitively activate FM identity. It may be that these meristems are gradually transitioning through a phase of mixed IM/FM identity in which they first initiate lateral organs that have sepal morphology but have the elongated internodes typical of bracts. Then, even when they express enough FM identity to generate whorls of sepals, the meristems are getting “stuck” on first whorl production such that it takes several whorls before they can move on to the rest of the floral organs. Based on organ counts in *Aquilegia*, it appears that the extra sepals are not due to a shift in homeotic gene expression but, rather, the intercalation of additional sepal whorls. In *Eschscholzia*, where Wreath et al. observed more widespread homeotic phenotypes and variation in organ number (see The AqUFO Paralogs Appear to Promote Petal and Stamen Initiation), it is less clear whether the additional calyx whorls were due to intercalation or homeosis. However, the presence in both taxa of internodal elongation between individual sepals or outer sepal whorls is a clear indicator of mixed meristem identity that supports this alternative interpretation. The observation of similar phenotypes in *Aquilegia* and *Eschscholzia* suggests that, across the Ranunulales, *LFY* and *UFO* homologs may primarily function to reinforce the switch from IM to FM identity in these cymose inflorescences, such that it is discrete and complete.

Silencing of *AqLFY* or *AqUFO1/2* does not eliminate FM identity, but in their absence, the switch becomes sloppy and gradual. This is reminiscent of the feed-forward loop that appears to act downstream of LFY in *Arabidopsis* (Saddic et al., 2006). Not only does LFY activate the floral organ identity genes, it also up-regulates multiple other FM identity loci, such as *AP1*, thereby promoting a robust switch to FM identity. Some of these additional FM identity genes function primarily downstream of LFY while others are also activated by as yet unidentified players. It is possible that in the Ranunculales, the nature of this feed-forward loop has been rewired such that *LFY* and *UFO* play more minor roles in FM identity itself but are still critical to the decisive activation of the FM identity program. Of course, it would also be consistent with what we know from other taxa if *AqLFY* is important for FM identity, but there are simply redundant loci that can function in its absence. As mentioned above, in *Aquilegia* the closest homolog of *AP1*, *AqFL1*, does not play this role since its silencing phenotype suggests a function in IM, rather than FM, identity (Pabon-Mora et al., 2013). It is still possible that *Aquilegia* FM identity loci include homologs of other known factors, such as *APETALA2* (reviewed Litt and Kramer, 2010) or *LATE MERISTEM IDENTITY1* (Saddic et al., 2006), or there may be completely novel players.

### The *AqUFO* Paralogs Appear to Promote Petal and Stamen Initiation

Another intriguing aspect of the observed phenotypes is the differences between the *aqlfy* vs. *aqufo* plants. While the traits we interpret as related to defects in FM identity (bract/sepal chimeras and additional sepal whorls) are shared among all the cohorts, targeted silencing of the *AqUFO* loci had a much stronger impact on both the presence of petals and the number of stamens. Interpreting this phenotype has several challenges, including the variable nature of VIGS itself and the fact that *AqLFY* expression is also reduced in *aqufo* flowers. The implication would be that the phenotypic differences between the *aqlfy* and *aqufo* flowers reflect functions that are specific to *AqUFO1/2* and independent of *AqLFY*. Based on what we know about *UFO* homolog function in other taxa, there are at least two potential contributing factors to these phenotypes.

First, *ufo* mutants commonly exhibit petal-to-sepal and stamen-to-carpel homeosis, consistent with the role of UFO in activating transcription of the B class genes with LFY (Lee et al., 1997). In our flowers, we did not observe stamen-to-carpel homeosis, but the loss of petals could be due in part to petal-to-sepal transformation. Further, such transformation could also explain the increased sepal number in *aqufo1*. The specific effect on petal identity rather than B function writ large could be most easily explained by positing that the *AqUFO1/2* paralogs are specifically required for the petal-specific paralog *AqAP3-3* (Sharma et al., 2011), which could simultaneously help explain the differential expression of this paralog relative to the other *AP3* loci. However, it is curious that this function would be independent of AqLFY.

Second, we must also consider the reduction of stamen numbers in the *AqUFO*-silenced cohorts. The clear reduction in overall organ numbers suggests that this is not due to homeosis but, rather, reduction in the number of initiated whorls. Previous studies of *UFO* in *A. thaliana* have shown that the gene plays separable roles in 1) the identity of petals and stamens, and 2) the initiation of second whorl organs (Durfee et al., 2003; Laufs et al., 2003). In *Aquilegia*, we observe that *AqUFO1* expression is associated not only with the petal whorl, but also the outer stamen whorls (**Figures 5B–D**), and *AqUFO2* is expressed in all initiating stamen primordia (**Figures 5E–F**). This raises the possibility that one or both of these loci are important for the initiation of petals and the outer whorls of stamens, in what may be an *AqLFY*-independent manner. In summary, there are several potential explanations for the extra sepal whorls and the loss of petals and stamens observed in the *aqufo* flowers relative to *aqlfy* flowers: 1) a loss of *AqAP3-3* expression leading to petal-to-sepal transformation, and/or 2) the combined effects of a loss of FM identity that produces extra sepal whorls and the deletion of petal and outer stamen whorls.

Regardless, there is clear evidence that *aqufo* impacts stamen numbers in a manner that is not observed in *aqlfy*. In *A. thaliana*, the loss of petal initiation in alleles such as *ufo-11* appears to depend on *LFY* function (Durfee et al., 2003), but it is also possible that this role is simply dependent on FM identity such that *lfy* is epistatic. By contrast, in *Aquilegia*, *aqlfy* still retains substantial FM identity, so *AqUFO* function in stamen (and possibly petal) initiation could be independent of *AqLFY*. It is interestingly to note in this regard that *EscFLO* silencing in *Eschscholzia* also results in reduced organ number in some flowers, particularly the stamens (Wreath et al., 2013). This observation, combined with the fact that we see reduced *AqLFY* expression in the *aqufo* flowers and have confirmed the ability of AqLFY to interact with at least AqUFO1, makes us hesitant to assert that the organ loss phenotype is definitively independent of *AqLFY*.

### Conclusions

The phenotypes recovered in our silencing of *AqLFY* and *AqUFO1/2* are complex and somewhat difficult to interpret, for a variety of reasons. There are several definitive conclusions that we believe can be made. First, *AqLFY* and *AqUFO1/2* promote the complete, precise transition of IM to FM identity such that in their absence, meristems progress through several nodes with mixed IM/FM identity before finally committing to FM fate. Second, there is little evidence for an essential role for *AqLFY* or *AqUFO1/2* in the activation of the B and C class genes, although a specific function in *AqAP3-3* expression may be possible, which could help explain the distinct expression of this paralog. Finally, the *AqUFO1/2* paralogs appear to promote the initiation of outer stamen whorls, and possibly the petals. This function is more sensitive to the targeted silencing of *AqUFO1/2*, but we cannot rule out a joint role for *AqLFY*. Many of these functions are consistent with what has been observed for *EscFLO*-silencing in the poppy *Eschscholzia californica*, suggesting that this model for *LFY* homolog function is conserved across the Ranunculales. Obviously, there are many remaining questions, perhaps the most pressing of which is, If *LFY* is not essential to FM identity in the Ranunculales, what is?

## Data Availability Statement

The F-box phylogeny dataset analyzed for this study can be found at https://kramerlab.oeb.harvard.edu/data-resources.

## Author Contributions

BS and EK coordinated and conceived of the study. BS prepared the TRV2 constructs and performed the VIGS treatments. BS and CM documented the phenotypes. CM conducted the *AqUFO1* and *AqUFO2 in situ* hybridization. BS and DW conducted the RT-qPCR analyses. CW-C prepared the Y2H constructs and LH performed the Y2H experiment. BS and EK interpreted the data, and drafted, revised, and approved the final article. All authors read and approved the final manuscript.

## Funding

This work was supported by the National Science Foundation (Grant No. IOS-1121005 to EK) and California State University, including the CSU-LSAMP program to DW and California State Polytechnic, Pomona to BS.

## Conflict of Interest

The authors declare that the research was conducted in the absence of any commercial or financial relationships that could be construed as a potential conflict of interest.
