# Supplementary material for: Homologs of LEAFY and UNUSUAL FLORAL ORGANS Promote the Transition From Inflorescence to Floral Meristem Identity in the Cymose Aquilegia coerulea
Source: Front Plant Sci. 2019 Oct 4;10:1218. doi: 10.3389/fpls.2019.01218 (PMC6805967; doi:10.3389/fpls.2019.01218)
Supplement: Supplementary file 1 [file DataSheet_1.pdf]

Supplementary Material

1 Supplementary Figures and Tables

1.1 Supplementary Figures

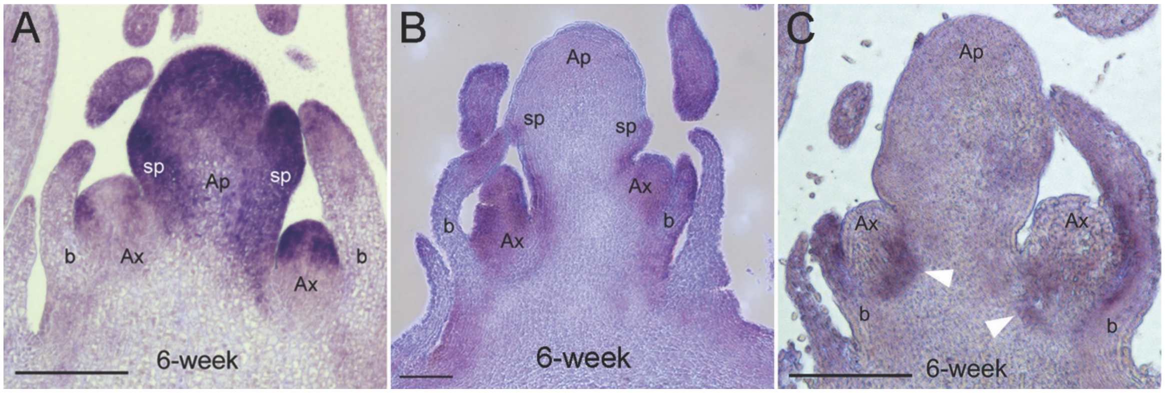

Supplementary Figure 1. Expression patterns of putative inflorescence meristem (IM) and floral meristem (FM) identity genes in the apices of *Aquilegia* plants 6 weeks into vernalization treatment, modified from Ballerini and Kramer (2011). A. *AqLFY* expression. The apical meristem (Ap) has undergone the transition to FM identity and is initiating sepals (sp). The associated axillary meristems (Ax), each subtended by their own bract (b) are in different developmental stages. The axillary meristem on the left appears to be in the IM phase, which lacks constitutive *AqLFY* expression, while the axillary meristem on the right may be just entering the floral identity phase with near constitutive *AqLFY* expression. B. *AqAGL24.2* expression. Expression appears to be constitutive in the axillary meristems (Ax), but the apical meristem (Ap), which is just beginning to initiate sepals (sp), has lost most *AqAGL24.2* expression. C. *AqTFL* expression. Expression is observed in a wedge-shaped domain associated with the inner base of the axillary meristems (white arrowheads). Also, some signal appears to be present in the bracts. No expression is seen in the terminal meristem (Ap), which has most likely transitioned to FM identity. Scale bar in A = 200 µm, in B = 100 µm, and in C = 50 µm.

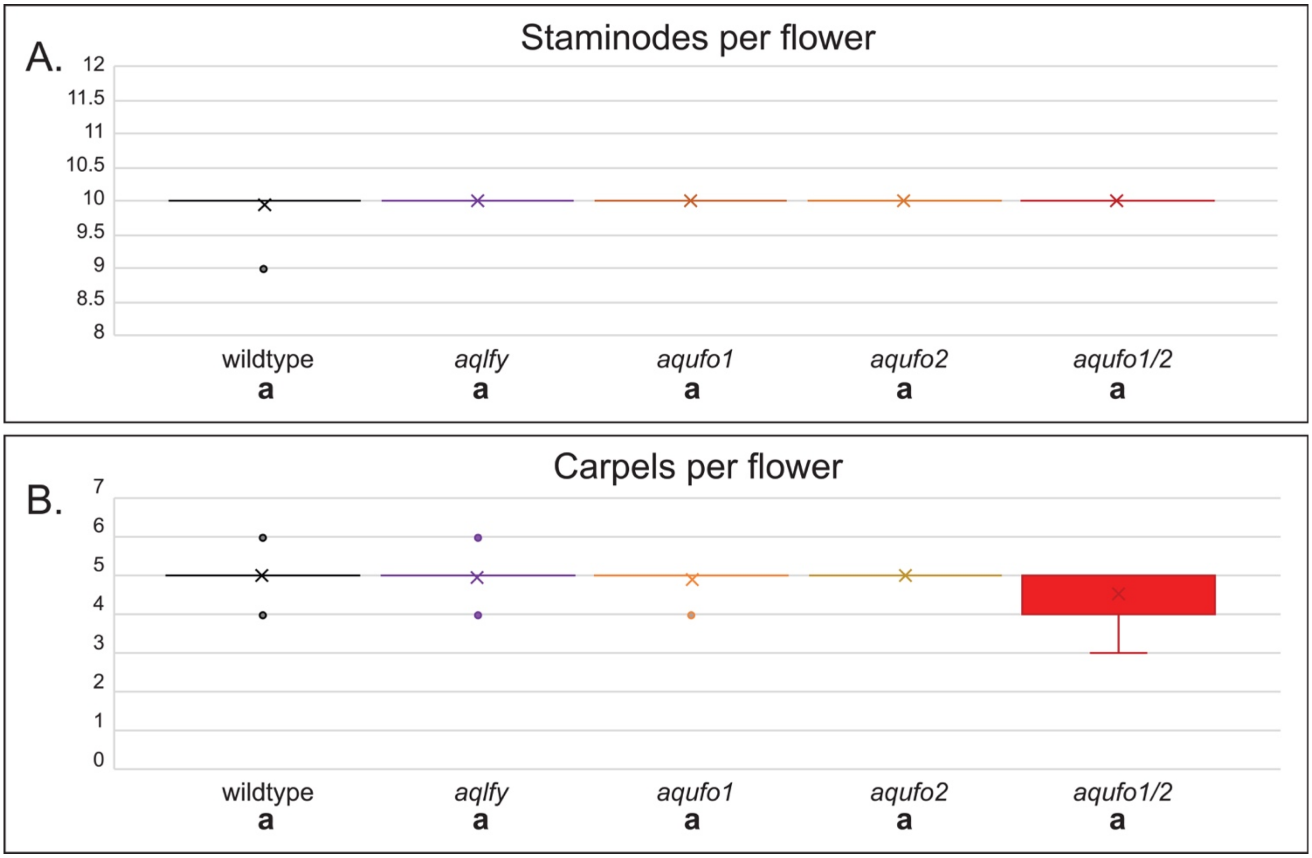

Supplementary Figure S2. Organ counts for wildtype and silenced flowers. Flowers were dissected from wildtype (*AqANS*-silenced control, n=15), *aqlfy* (n=38), *aqufo1* (n=71), *aqufo2* (n=32), and *aqufo1/2* (n=25) cohorts. The per flower distributions above are presented for staminodes (A) and carpels (B), while the other organ counts are presented in Fig. 3. For each class of data (A-B), a one-way ANOVA was conducted to determine whether any of the means were statistically different. For these organ classes, there was no difference between the cohorts, as indicated by the "a" labels.

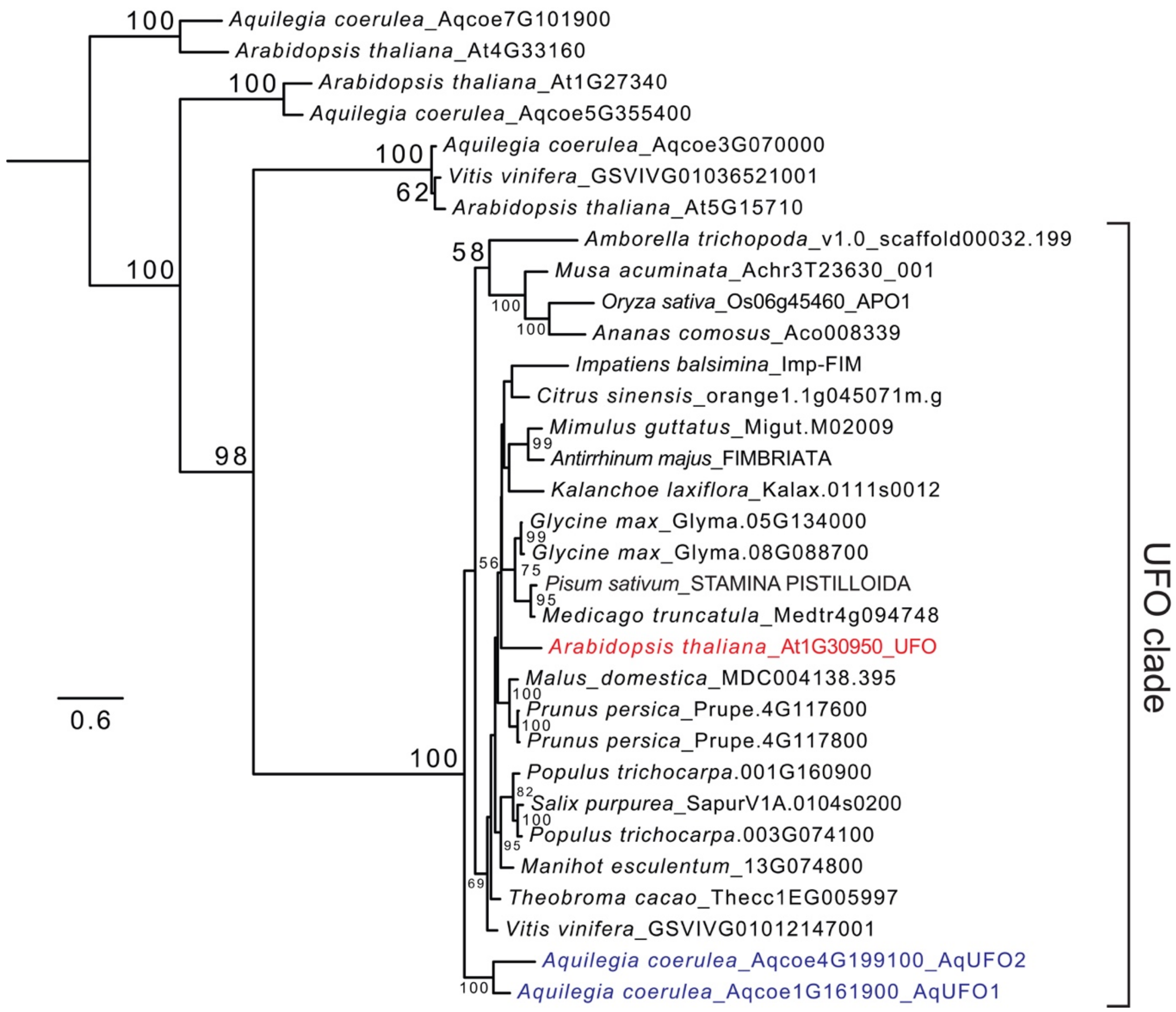

Supplementary Figure S3. Maximum likelihood tree showing relationships among amino acids sequences encoded by 25 putative *UFO* homologs and three outgroup lineages drawn from the C5 clade of the F-box superfamily (Gagne et al., 2002). Bootstrap values greater than 50 are shown at the nodes. The vast majority of these loci are drawn from the Phytozome database, with the exception of several previously characterized *UFO* orthologs drawn from the following publications: *Arabidopsis thaliana* UFO (highlighted in red), (Samach et al., 1999); *Pisum sativum* STAMINA PISTILLOIDA, (Taylor et al., 2001); *Antirrhinum majus*, FIMBRIATA, (Simon et al., 1994); *Impatiens balsimina* Imp-FIM, (Pouteau et al., 1998); and *Oryza sativa* APO1, (Ikeda-Kawakatsu et al., 2009). The clade termed the UFO clade has 100% bootstrap support and includes both *Aquilegia* loci in the current study.

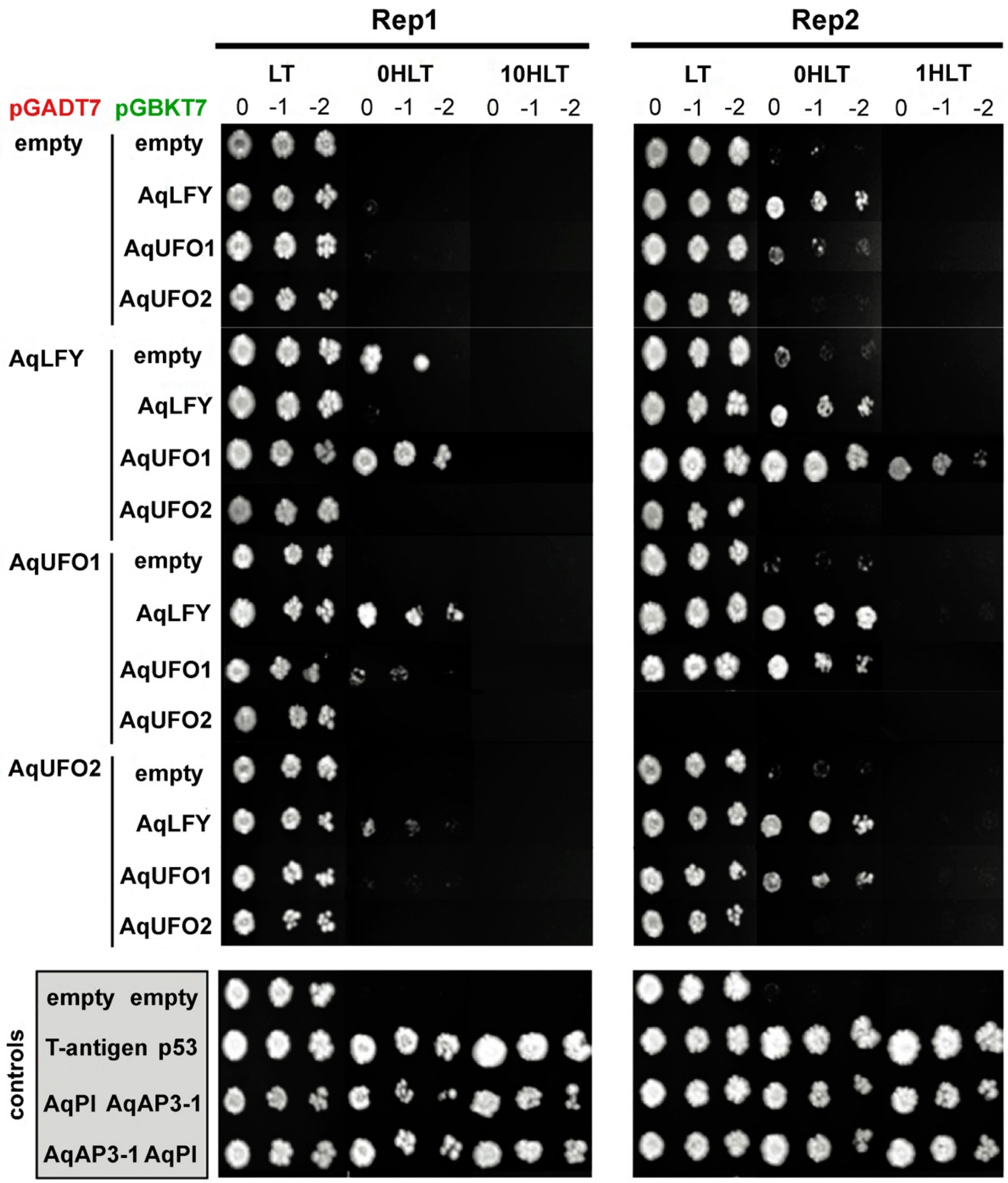

Supplementary Figure 4. Test for protein-protein interactions between AqLFY, AqUFO1, and AqUFO2 in AH109 yeast. Replicate experiments (Rep1 and Rep2) of yeast two-hybrid assays were conducted to test for dimerization between N-terminal GAL4AD fusions of AqLFY, AqUFO1, AqUFO2 in the activation domain vector (pGADT7, left column) and N-terminal GAL4BD fusions of same proteins in the binding domain vector (pGBKT7, right column). The observed growth after 6 days on either nonselective (SD dropout media lacking leucine and tryptophan (LT)) and restrictive (SD dropout media lacking leucine, tryptophan, and histidine (HLT)) is presented. It appears that AqLFY/AqUFO1, AqLFY/AqUFO2, and UFO1/UFO2 could form heterodimers in this system. AqLFY dimerizes with AqUFO1 more strongly than with AqUFO2, as indicated by growth of yeast containing AqLFY-AqUFO1 with addition of 1mM histidine synthesis inhibitor 3-amino-1, 2, 4-triazole (3-AT), right photo panel. Some homodimerization was also detected for AqLFY and

AqUFO1. Note that Rep1 and Rep2 tested growth on restrictive HLT media with addition of either 10 or 1 mM 3-AT, respectively. Autoactivation only occurred when AH109 contained the BD construct with AqLFY, but growth was minimized with the addition of 1 mM 3-AT to the SD HLT media. The manufacturer's positive control (p53/T-antigen) was used to monitor Y2H assays as well as two *Aquilegia* MADS proteins (AqAP31, AqPI) known for their strong protein-protein interaction (Kramer et al., 2007). Note that the AqUFO1/AqUFO2 heterodimer culture did not grow in Rep2.

1.2 Supplementary Table

Supplementary Table 1: PCR Primers

**Primer name**	**Primer seq**	**Fragment size**
In situ Probe Primers		
AqUFO1 F	5’ ATATGTCATGGTAGCAGGTGACGATCAA	323 bp
AqUFO1 R	5’ GAATCACTTGGCAACCTCCACACTCT	
AqUFO2 F	5’ ACGATCACCCAGTTTAGTGGAATGTAGA	348 bp
AqUFO2 R	5’ CCTGCAATCCATCACCACCATAGA	
VIGS Construct Primers		
AqLFY F-BamHI	5’ AAGGATCCACGTGGTTGTGGTCGTCATA	532 bp
AqLFY R-Kpn	5’ TAGGTACCACTAGATCAGAATGAAGCTGAGTT	
AqUFO1 F-EcoRI	5’ CGGAATTCGCAACCCAGTTTCCAAATTATTCAC	359 bp
AqUFO1 R-XbaI	5’ ACTCTAGATTTCATTGGTGGCTCCATTTCGC	
AqUFO2 F BamHI	5’ AAGGATCCGCAACCCAGTCTCCAAATCTCTCAT	389 bp
AqUFO2 R-Kpn	5’ TAGGTACCTCTACATTCCACTAAACTGGGTGATCGTAG	
qRT-PCR Primers		
AqLFY rtF	5’ ACACCACTAACCCTCTTGACGC	174 bp
AqLFY rtR	5’ TTTTCATCTCCGCTATTCCTCG	
AqUFO1 rtF	5’ ATGCTTTGTTGAGGATTACTATCTCCG	231 bp

AqUFO1 rtR	5' ACAATTAAACCACCAGAGGAAGCAGC	
AqUFO2 rtF	5' TGATTTGTATCTTCACATTGCACCA	217 bp
AqUFO2 rtR	5' TTATTAACCCAGCAGAGGAAGCGGC	
Yeast Two-Hybrid Primers		
AqLFY F	5' CCGAATTCATGGATCCAGAAGCATTCTCAGCAG	
AqLFY R	5' CCGAATTCGAATGAAGCTGAGTTTGAAGTAGAGG	
AqUFO1 F	5' CCGAATTCATGGAGCAACATATCAACATGTTTA	
AqUFO2 R	5' CCGAATTCGTAGTGATAATTAGCCATTGAAGCTT	
AqUFO2 F	5' CCGAATTCATGGATCCTGAGATATGGAGCAGAC	
AqUFO2 R	5' CCGAATTCAGGAAATGCTGATTGACTAAATAGATCCA	

References Cited

Ballerini, E.S., and Kramer, E.M. (2011). The control of flowering time in the lower eudicot *Aquilegia formosa*. *EvoDevo* 2**,** 4.

Gagne, J.M., Downes, B.P., Shiu, S.-H., Durski, A.M., and Vierstra, R.D. (2002). The F-box subunit of the SCF E3 complex is encoded by a diverse subfamily of genes in Arabidopsis. *Proc Nat'l Acad Sci, USA* 99**,** 11519-11524.

Ikeda-Kawakatsu, K., Yasuno, N., Oikawa, T., Iida, S., Nagato, Y., Maekawa, M., and Kyozuka, J. (2009). Expression Level of ABERRANT PANICLE ORGANIZATION1 Determines Rice Inflorescence Form through Control of Cell Proliferation in the Meristem. *Plant Physiology* 150**,** 736-747.

Kramer, E.M., Holappa, L., Gould, B., Jaramillo, M.A., Setnikov, D., and Santiago, P. (2007). Elaboration of B gene function to include the identity of novel floral organs in the lower eudicot Aquilegia (Ranunculaceae). *Plant Cell* 19**,** 750-766.

Pouteau, S., Nicholls, D., Tooke, F., Coen, E., and Battey, N. (1998). Transcription pattern of a FIM homologue in Impatiens during floral development and reversion. *Plant J* 14**,** 235-246.

Samach, A., Klenz, J.E., Kohalmi, S.E., Risseeuw, E., Haughn, G.W., and Crosby, W.L. (1999). The UNUSUAL FLORAL ORGANS gene of Arabidopsis thaliana is an F-box protein required for normal patterning and growth in the floral meristem. *Plant J* 20**,** 433-445.

Simon, R., Carpenter, R., Doyle, S., and Coen, E. (1994). Fimbriata controls flower development by mediating between meristem and organ identity genes. *Cell* 78**,** 99-107.

Taylor, S., Hofer, J., and Murfet, I. (2001). Stamina pistilloida, the pea ortholog of Fim and UFO, is required for normal development of flowers, inflorescences, and leaves. *Plant Cell* 13**,** 31-46.
